# Veterinary Association of Manitoba

**Published:** 1896-05

**Authors:** 


### VETERINARY ASSOCIATION OF MANITOBA.

THE annual meeting was held in Delmonico's Hall, Winnipeg, on the 20th and 21st of February last. The members present were Drs. Young, Manitou; Swenerton, Wawanesa; Ward, Selkirk; McNaught, Rapid City; McFadden, Emerson; Rutherford, Portage les Prairie; McGillivray, Manitou; Hinman, Little, Murray, and Dunbar, Winnipeg.

The following members constitute the Council for the current year: Drs. Rutherford, Torrance, Young, Thompson, Hinman, Swenerton, and Dunbar. The Council met and elected the following officers: President, Dr. Young, Manitou; Vice-President, Dr. Swenerton, Wawanesa; Secretary-Treasurer and Registrar, Dr. Dunbar, Winnipeg. Drs. Rutherford, Hinman, and Dunbar were elected examiners.

After the usual routine business, the time was devoted to the discussion of subjects of interest to the profession. Dr. McNaught exhibited a very ingenious truss for supporting a poultice applied to the knee or hock of a horse. Dr. Rutherford described, in a very interesting manner, a peculiar case of a tumor, weighing six pounds, of the osteo-fibroma type, which he found deeply imbedded in the muscular tissue of a mare's leg. Other uncommon and interesting cases were related by Drs. Hinman, Swenerton, and Dunbar.

Dr. Ward read an instructive and carefully prepared paper on "The Hereditary Transmission of Tuberculosis," which was followed by a protracted and profitable discussion of the subject, participated in by Drs. Rutherford, Hinman, McNaught, and Dunbar. Dr. Wright, of Grenfell, N. W. T., who was present as a visitor, also took part in the discussion, and by his remarks contributed not a little to the interest of the meeting. A resolution was passed recommending the tuberculin-test for tuberculosis, as being a valuable diagnostic agent.

The meeting throughout was pleasant and highly edifying, and afforded ample testimony that one of the chief objects of the Association is being accomplished, namely, the elevation of the standard of the veterinary profession in Manitoba.

The semi-annual meeting of the Association will be held in Brandon in July.

